# Cadmium exposure and age-associated DNA methylation changes in non-smoking women from northern Thailand

**DOI:** 10.1093/eep/dvx006

**Published:** 2017-07-18

**Authors:** Kathryn Demanelis, Shama Virani, Justin A. Colacino, Niladri Basu, Muneko Nishijo, Werawan Ruangyuttikarn, Witaya Swaddiwudhipong, Kowit Nambunmee, Laura S. Rozek

**Affiliations:** 1 Department of Environmental Health Sciences, University of Michigan, Ann Arbor, MI 48104, USA; 2 Faculty of Agricultural and Environmental Sciences, McGill University, Montreal, QC, H9X3V9, Canada; 3 Department of Public Health, Kanazawa Medical University Hospital, Uchinada, 920-0293, Ishikawa, Japan; 4 Department of Forensic Medicine, Faculty of Medicine, Chiang Mai University, Chiang Mai 50200, Thailand; 5 Department of Community and Social Science, Mae Sot General Hospital, Mae Sot District, Tak Province 63110, Thailand; 6 School of Health Science, Mae Fah Luang University, Chiang Rai 57100, Thailand

**Keywords:** cadmium, biologic aging, DNA methylation, thailand, epigenomics, toxic metals

## Abstract

DNA methylation changes with age, and may serve as a biomarker of aging. Cadmium (Cd) modifies cellular processes that promote aging and disrupts methylation globally. Whether Cd modifies aging processes by influencing establishment of age-associated methylation marks is currently unknown. In this pilot study, we characterized methylation profiles in > 450 000 CpG sites in 40 non-smoking women (age 40–80) differentially exposed to environmental Cd from Thailand. Based on specific gravity adjusted urinary Cd, we classified them as high (HE) and low (LE) exposed and age-matched within 5 years. Urinary Cd was defined as below 2 µg/l in the LE group. We predicted epigenetic age (DNAm-age) using two published methods by Horvath and Hannum and examined the difference between epigenetic age and chronologic age (Δage). We assessed differences by Cd exposure using linear mixed models adjusted for estimated white blood cell proportions, BMI, and urinary creatinine. We identified 213 age-associated CpG sites in our population (*P* < 10^−4^). Counterintuitively, the mean Δage was smaller in HE vs. LE (Hannum: 3.6 vs. 7.6 years, *P* = 0.0093; Horvath: 2.4 vs. 4.5 years, *P* = 0.1308). The Cd exposed group was associated with changes in methylation (*P* < 0.05) at 12, 8, and 20 age-associated sites identified in our population, Hannum, and Horvath. From the results of this pilot study, elevated Cd exposure is associated with methylation changes at age-associated sites and smaller differences between DNAm-age and chronologic age, in contrast to expected age-accelerating effects. Cd may modify epigenetic aging, and biomarkers of aging warrant further investigation when examining Cd and its relationship with chronic disease and mortality.

## Introduction

The epigenetic clock is hypothesized to capture common and progressive changes in methylation that occur during aging at specific CpG sites that are susceptible to the decline in maintaining methylation patterns [1, 2]. DNA methylation can affect downstream gene expression, and it is a mechanism that is dynamic and sensitive to internal and external environmental changes that result from aging, lifestyle factors, disease, and exposure to environmental toxicants [3]. DNA methylation becomes increasingly dysregulated with age, and aging is associated with loss of global methylation and preferential gains in methylation at island-associated promoters [4–6]. Using human methylation arrays, several studies have shown that a combination of methylation at CpG sites can describe an epigenetic clock and predict epigenetic age [1, 4, 7, 8]. Multiple studies have characterized that greater difference between chronologic age and epigenetic age is associated with health and mortality [9, 10]. Environmental exposures that alter the epigenome may modify the rate at which alterations in DNA methylation accrue during aging.

Cadmium (Cd) is a toxic metal, and environmental exposure is associated with changes in the cellular environment, including epigenomic changes, and potentially, with health in later life [11]. Cd initiates aging-related cellular processes, including perturbation of gene-specific and global methylation. DNA Methylation may be sensitive to Cd-associated changes in the oxidative [12, 13] and inflammatory environment of the cell [14]. Cd is also associated with altered DNA methyltransferase activity as suggested by in vivo and in vitro studies [15–17].

The relationship between Cd exposure and age-associated methylation changes has not been thoroughly investigated in an environmentally exposed population. We have previously described DNA methylation changes in a population in northern Thailand that was environmentally exposed to Cd [18]. The population was exposed to Cd after ingesting contaminated rice and water resulting from downstream environmental pollution from nearby zinc mines [19]. This population has documented levels of urinary Cd levels that exceed the WHO standard level [20] and associated chronic health problems such as diabetes, hypertension, and osteoporosis [21, 22]. In the current work we conducted a pilot study of non-smoking women from northern Thailand with high and low levels of Cd to evaluate the cross-sectional relationship among age, DNA methylation, and Cd exposure using epigenome-wide data and hypothesized that increased Cd exposure is associated with increased epigenetic age acceleration.

## Results

### Study Population Characteristics

The median and range of SG-adjusted Cd was 0.7 µg/l (0.3–1.9 µg/l) in the low Cd exposure (LE) group and 10.8 µg/l (7.0–48.3 µg/l) in the high Cd exposure (HE) group. The mean age in the (LE) and HE groups were 58.8 (SD=9.4) and 60.4 (SD=10.3) years (Table 1). The LE had significantly higher BMI than the HE group, 26.4 vs. 22.2 kg/m^2^ (*P* = 0.0028).

**Table 1:** descriptive statistics of study population stratified by exposure group

	Low Cd (*n* = 20)	High Cd (*n* = 20)	*P*	Spearman correlation age	*P*	Spearman correlation adjusted Cd	*P*
Adjusted Cd (µg/l)	0.71 (0.26, 1.94)	10.76 (6.97, 48.3)	**<0.0001**	0.204	0.2061		
Age (years)	58.8 (9.4)	60.4 (10.3)	0.6106			0.204	0.2061
BMI (kg/m^2^)	26.4 (2.9)	22.2 (4.9)	**0.0024**	−0.246	0.1263	−0.387	**0.0137**
Adjusted urinary markers							
Urinary Creatinine (µg/l)	0.91 (0.47, 1.67)	1.29 (0.65, 2.00)	**0.0016**	−0.203	0.2119	0.398	**0.0115**
Urinary beta-2 microglobulin (µg/mL)	0.09 (0.02, 17.22)	5.26 (0.15, 40.50)	**<0.0001**	0.274	0.0920	0.674	**<0.0001**
Urinary NAG (µg/l)	4.00 (1.31, 26.10)	9.25 (2.84, 30.50)	**0.0036**	0.187	0.2486	0.444	**0.0041**
Urinary citrate (µg/l)	0.31 (0.09, 0.55)	0.09 (0.02, 0.28)	**<0.0001**	−0.112	0.4926	−0.628	**<0.0001**
Blood counts							
White blood cell count (10^6^ cells/µl)	9.47 (3.87)	7.80 (1.89)	0.0938	0.011	0.9465	−0.290	0.0693
Hemoglobin (g/dl)	12.94 (1.02)	12.43 (1.13)	0.1380	−0.056	0.7316	−0.238	0.1400
Hematocrit (%)	39.85 (3.32)	37.57 (3.20)	**0.0334**	−0.073	0.6567	−0.337	**0.0333**
Red Blood Cell Count (10^6^ cells/µl)	4.85 (0.66)	4.44 (0.57)	**0.0409**	−0.191	0.2379	−0.396	**0.0115**
Estimated white blood cell proportions							
CD8T (%)	8.28 (4.26)	7.15 (4.84)	0.4339	0.013	0.9372	−0.199	0.2169
Granulocyte (%)	51.92 (9.24)	51.43 (7.81)	0.8581	−0.273	0.0884	0.007	0.9645
Monocyte (%)	4.52 (2.09)	6.48 (2.88)	**0.0185**	−0.093	0.5689	0.441	**0.0047**
CD4T (%)	10.19 (4.13)	9.81 (4.87)	0.7950	0.076	0.6423	−0.053	0.7424
Natural Killer (%)	17.11 (6.76)	18.39 (7.23)	0.5669	0.278	0.0833	0.164	0.3114
B Cells (%)	6.01 (3.24)	3.82 (3.03)	**0.0334**	0.163	0.3141	−0.267	0.0963

Non-smoking northern Thai women age 40–80 with high (*n* = 20) and low (*n* = 20) exposure to Cd. Medians and ranges are reported for adjusted urinary Cd and urinary markers, which were non-normally distributed, and *P*-values from Wilcoxon sum rank test were reported. Mean and SDs are reported for all other covariates, which were normally distributed, and *P*-values from *t*-tests were reported. Spearman correlations were computed for each covariate and its relationship with age and adjusted Cd and corresponding *P*-values were reported.

Urine SG was not significantly different between exposure groups. The mean urinary creatinine was significantly higher in the HE compared with the LE group, 1.29 vs. 0.91 µg/l, respectively (*P* = 0.0016). Both urinary beta-2-microglobulin, a biomarker of chronic kidney disease, and N-acetyl-beta-D-glucosaminidase, a biomarker of renal tubular impairment were significantly elevated in the HE compared with LE (*P* < 0.0001 and *P* = 0.0036, respectively). Urinary citrate, a biomarker of renal stone formation, was significantly lower in the HE compared with LE (*P* < 0.0001). These results among our study population are consistent with renal damage identified among Mae Sot [23]. Hematocrit and total red blood cell count were also, on average, lower in the HE group (*P* = 0.03 and 0.04, respectively). Further, HE individuals had an increased estimated percentage of monocytes (*P* = 0.02) and decreased B cells (*P* = 0.03).

### DNAm-Age and Association with Cd Exposure

**Figure 1:**
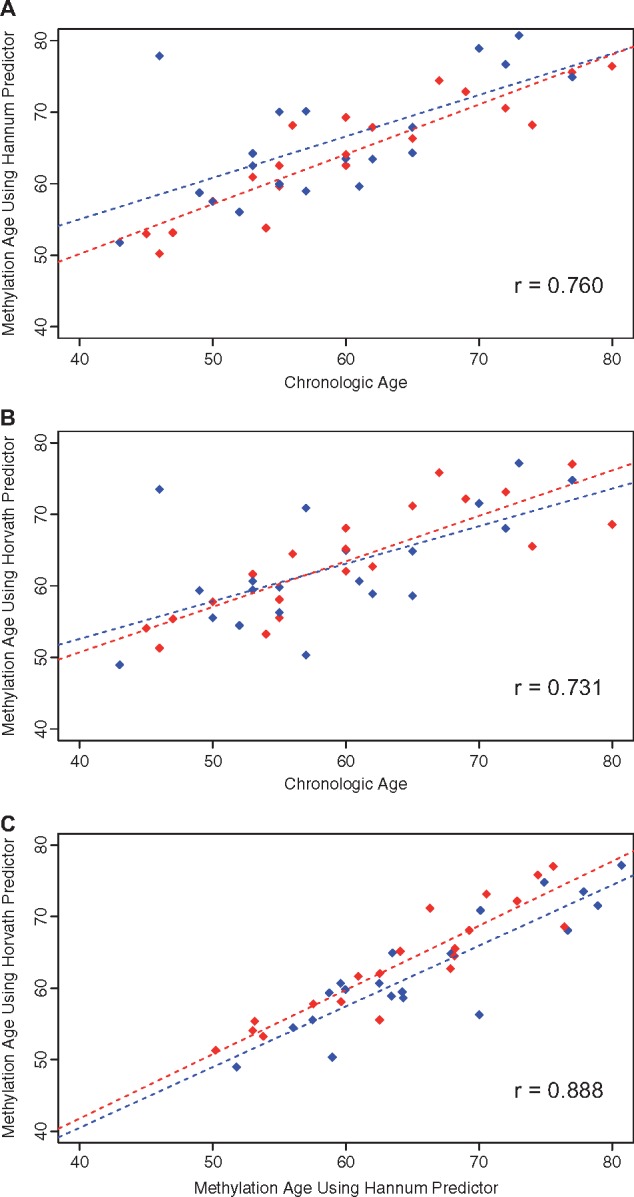
Hannum and Horvath Methylation Age (DNAm-age). Blue and red denotes LE (*n* = 20) and HE (*n* = 20) groups, respectively. Scatterplots are presented for Hannum DNAm-age vs. chronologic age **(A)**, Horvath DNAm-age vs. chronologic age **(B)**, and Horvath DNAm-age vs. Hannum DNAm-age **(C)**. Spearman correlation coefficients are reported for entire study population (*P* < 0.0001 for each)

The Hannum and Horvath DNAm-ages positively and significantly correlated with chronologic age, 0.76 (*P* < 0.0001) and 0.73 (*P* < 0.0001), respectively (Fig. 1). The DNAm-age from Hannum and Horvath were also positively and significantly correlated with each other, 0.88 (*P* < 0.0001). Hannum and Horvath DNAm-ages were on average 5.56 (SD = 6.37) and 3.49 (SD = 6.70) years greater than chronologic age, respectively.

The mean DNAm-age did not significantly differ by exposure group [Hannum: LE = 65.9 (SD = 8.4) and HE = 64.4 (SD = 8.0) years; Horvath: LE = 62.4 (SD = 8.0) and HE = 63.7 (SD = 7.8) years]. Among the LE group, the Spearman correlation between Hannum and Horvath DNAm-age and chronologic age was 0.63 (*P* = 0.0029) and 0.55 (*P* = 0.0128), respectively, while among the HE group, the correlations were 0.93 (*P* < 0.0001) and 0.89 (*P* < 0.0001), respectively (Fig. 1). Within the HE group, the Hannum and Horvath DNAm-age increased by 0.69 (*P* < 0.0001, *r*^2^ = 0.804) years and 0.64 years (*P* < 0.0001, *r*^2^ = 0.692) per year in chronologic age, respectively. Although in the LE group, the Hannum and Horvath DNAm-age significantly increased by 0.58 years (*P* = 0.0021, *r*^2^ = 0.384) and 0.53 years (*P* = 0.0042, *r*^2^ = 0.339) per year in chronologic age, respectively.

**Figure 2:**
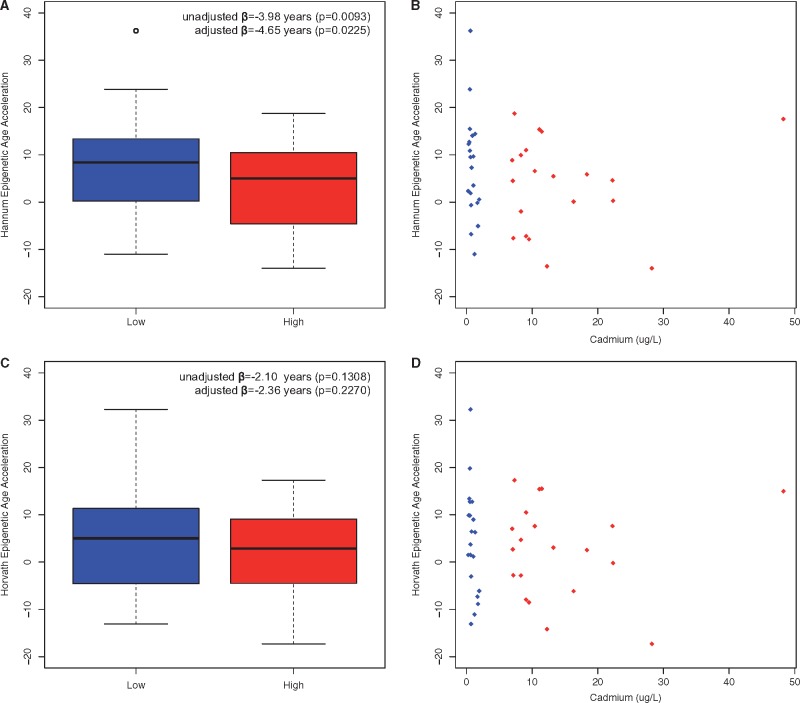
epigenetic age acceleration (Δage). Epigenetic age acceleration (Δage) is calculated as the difference between adjusted DNAm-age and chronologic age. Boxplots of Δage are presented for Hannum **(A)** and Horvath **(C)**. Relationship between Cd (µg/l) and Δage are presented for Hannum **(B)** and Horvath **(D)**. Blue and red denote LE and HE group, respectively

The mean Δage using Hannum was 7.6 years (SD = 10.9 years) in the LE group and 3.6 years (SD = 9.9 years) in the HE group. Counterintuitively, the Δage was significantly smaller in the HE group compared with LE group (unadjusted: β = −3.98 years, *P* = 0.0093; adjusted: β = −4.65 years, *P* = 0.0225) (Fig. 2). The mean Δage using Horvath was 4.5 years (SD = 11.2 years) in the LE group and 2.4 years (SD = 10.0 years) in the HE group. The Δage for Horvath was also smaller in the HE group compared with LE group (unadjusted: β = −2.10 years, *P* = 0.1308; adjusted: β = −2.36 years, *P* = 0.2270). In addition, Hannum Δage significantly decreased per percent increase in continuous Cd exposure (adjusted β = −0.015, *P* = 0.0311) while The Horvath Δage was not significantly associated with continuous Cd exposure (adjusted β = −0.010, *P* = 0.1035). No associations were found between Δage and our urinary biomarkers of kidney function after adjustment (not shown).

### Methylation among Sites Included in Hannum and Horvath Methylation Age Predictors

At a significance level of *P* = 0.05, 56% (*n* = 40) of CpG sites included in the Hannum methylation age predictor were correlated with age in our study population (not shown). After adjustment by estimated blood cells, BMI, and urinary creatinine, 61% (*n* = 43) of sites included in the Hannum predictor were associated with age. Only 14% (*n* = 51) of sites included in the Horvath predictor were correlated with age in our study population, and after adjustment, 15% (*n* = 53) of these sites were associated with age.

Among the Hannum sites, eight sites were associated (*P* < 0.05) with Cd exposure group after adjustment (Table 2). The difference in methylation among these eight sites was between 1.1 and 3.1% lower in the HE compared with the LE group, and among all of the Hannum sites, difference in methylation was lower in HE compared with LE group in 75% of the sites (*n* = 53). In addition, eight sites were associated (*P* < 0.05) with continuous Cd exposure after adjustment. Among the Horvath sites, 20 sites were associated (*P* < 0.05) with Cd exposure group after adjustment. Fourteen sites were associated with decreased methylation (range: −0.8 to −3.4%) in HE group compared with LE group while eleven sites were associated with increased methylation (range: 0.2–3.5%) in HE compared with LE group. Among all of the Horvath sites, difference in methylation was lower in HE compared with LE group in 59% of the sites (*n* = 210). Fourteen sites were associated (*P* < 0.05) with continuous Cd exposure after adjustment.

**Table 2:** results of age-associated methylation by cd exposure group

	Chr	Gene	Location	Relation to CpG Island	Mean (SD) % methylation: low	Mean (SD) % methylation: high	Change in methylation by exposure group after adjustment	*P*-value	Change in methylation per percent change in Cd	*P*
Hannum										
cg16054275	1	F5	TSS1500	Open Sea	41.1% (3.1%)	40.4% (3.6%)	–3.12%	3.46E-02	–0.010%	4.91E-02
cg02085953	2	ARID5A	TSS1500	North Shore	34.9% (2.7%)	34.9% (3.3%)	–2.07%	2.55E-02	–0.007%	2.90E-02
cg04474832	3	ABHD14A	TSS1500	North Shore	26.7% (2.2%)	26.0% (2.6%)	–2.16%	1.04E-02	–0.006%	4.21E-02
cg02650266	4			Island	11.0% (3.4%)	10.3% (3.7%)	–2.17%	2.72E-02	–0.007%	4.33E-02
cg23500537	5			Open Sea	45.5% (3.3%)	44.6% (4.1%)	–2.79%	1.68E-02	–0.009%	2.29E-02
cg06685111	6	HCG18	TSS1500	South Shore	39.9% (2.0%)	39.9% (1.7%)	–1.31%	5.95E-02	–0.005%	2.79E-02
cg07927379	7	RNF32	TSS1500	Island	3.7% (1.1%)	3.6% (1.0%)	–1.01%	1.44E-02	–0.004%	2.70E-03
cg02867102	17			Open Sea	8.3% (2.7%)	6.9% (1.7%)	–2.13%	1.65E-02	–0.006%	4.52E-02
cg14692377	17	SLC6A4	5’UTR	Island	16.7% (2.9%)	15.2% (3.3%)	–2.18%	4.31E-02	–0.007%	7.43E-02
Horvath										
cg10045881	1	CHI3L2	5’UTR	Open Sea	64.3% (3.0%)	64.3% (3.8%)	1.77%	1.01E-01	0.007%	4.49E-02
cg14992253	1	EIF3I	TSS1500	Island	16.3% (3.1%)	16.4% (3.4%)	3.13%	2.72E-02	0.012%	1.06E-02
cg21870884	1	GPR25	1^st^ Exon	Island	31.2% (2.9%)	30.9% (3.0%)	–1.51%	6.92E-02	–0.006%	3.62E-02
cg02275294	1	SOAT1	TSS1500	North Shore	10.1% (2.4%)	9.7% (2.4%)	–1.48%	3.90E-02	–0.005%	6.05E-02
cg04474832	3	ABHD14A	TSS1500	North Shore	26.7% (2.1%)	26.0% (2.6%)	–2.16%	1.04E-02	–0.006%	4.21E-02
cg14163776	3	ACAP2	TSS1500	South Shore	20.9% (4.0%)	20.5% (3.2%)	–2.54%	4.91E-02	–0.009%	3.52E-02
cg23092072	4	AFF1	TSS1500	North Shore	2.9% (0.3%)	2.8% (0.3%)	0.23%	4.95E-02	0.001%	8.42E-02
cg23941599	5	FEM1C	TSS1500	South Shore	13.5% (2.9%)	11.9% (3.9%)	–2.36%	1.37E-02	–0.007%	3.15E-02
cg16150435	6	C6orf15	TSS200	Open Sea	81.9% (3.2%)	81.7% (3.4%)	–2.70%	3.04E-02	–0.008%	7.10E-02
cg25070637	8	SDC2	TSS200	Island	5.5% (1.1%)	5.1% (0.8%)	–0.57%	7.63E-02	–0.003%	9.29E-03
cg02654291	9	C9orf64	TSS1500	Island	51.0% (5.0%)	49.5% (2.3%)	–3.10%	4.76E-02	–0.011%	5.17E-02
cg04094160	9	ZBTB5	TSS1500	Island	2.9% (0.3%)	2.8% (0.4%)	–0.24%	7.72E-02	–0.001%	1.79E-02
cg13547237	11	C11orf68	TSS1500	South Shore	29.8% (2.8%)	28.7% (2.8%)	–2.71%	3.30E-02	–0.008%	6.00E-02
cg19692710	11	DNAJB13	5'UTR	Open Sea	87.4% (2.6%)	87.5% (2.7%)	–1.91%	3.06E-02	–0.006%	5.90E-02
cg24058132	14	GALC	5’UTR	South Shore	44.1% (3.6%)	43.7% (3.6%)	–3.40%	4.45E-03	–0.010%	1.20E-02
cg22432269	15	CYFIP1	5’UTR	Island	2.8% (0.3%)	2.9% (0.4%)	0.37%	2.32E-02	0.001%	5.56E-02
cg27015931	16	C16orf65	5'UTR	Open Sea	11.1% (1.1%)	10.7% (1.2%)	–0.82%	4.67E-02	–0.002%	9.59E-02
cg25928579	17	HOX8B	TSS1500	South Shore	7.0% (1.4%)	7.6% (1.5%)	1.38%	1.33E-02	0.004%	2.33E-02
cg00091693	17	KRT20	TSS200	Open Sea	80.5% (3.6%)	79.5% (4.0%)	–2.30%	5.30E-02	–0.009%	2.09E-02
cg02580606	17	KRT33B	TSS1500	Open Sea	90.0% (1.6%)	89.8% (1.3%)	–1.49%	5.76E-03	–0.005%	5.70E-03
cg19478743	17	ZMYND15	TSS1500	Island	6.2% (1.9%)	5.4% (1.0%)	–1.65%	8.16E-03	0.002%	1.11E-01
cg10486998	18	GALR1	TSS1500	Island	13.8% (3.2%)	15.0% (3.1%)	3.14%	1.92E-02	0.007%	1.37E-01
cg13899108	19	PDE4C	5'UTR	South Shore	45.6% (3.9%)	49.2% (5.4%)	3.52%	3.39E-02	0.009%	1.06E-01
cg12830694	19	PPP1R14A	TSS1500	South Shore	92.6% (1.2%)	92.3% (1.3%)	–0.85%	5.69E-02	–0.004%	1.11E-02
cg22449114	20	TCF15	Body	Island	36.5% (2.8%)	35.8% (4.0%)	–2.37%	8.27E-02	–0.009%	4.34E-02
cg01407797	22	CCDC117	TSS200	North Shore	5.9% (1.2%)	5.5% (1.4%)	–1.18%	4.56E-02	–0.004%	5.42E-02
cg11932564	22	TNFRSF13C	Body	Island	2.5% (0.8%)	2.6% (1.1%)	–0.78%	3.94E-02	–0.002%	9.30E-02
Age-associated in population									
cg26985289	1	CLSTN1	TSS200	Island	7.3% (3.9%)	6.7% (2.6%)	–1.52%	3.62E-02	–0.005%	7.23E-02
cg01695225	2	EMX1	Body	South Shore	12.1% (2.2%)	11.1% (2.3%)	–1.65%	1.60E-02	–0.006%	9.08E-03
cg04562589	4			Open Sea	23.1% (2.8%)	21.6% (2.3%)	–1.68%	3.91E-02	–0.005%	1.04E-01
cg02044219	4	LGI2	TSS200	South Shore	6.6% (4.0%)	5.1% (1.7%)	–2.41%	4.64E-02	–0.007%	1.70E-01
cg23500537	5			Open Sea	45.5% (3.3%)	44.6% (4.1%)	–2.79%	1.68E-02	–0.009%	2.29E-02
cg16001722	6	C6orf174	Body	Island	13.8% (2.9%)	12.9% (2.9%)	–1.30%	3.15E-02	–0.005%	1.87E-02
cg03465320	6	TAP1	TSS1500	South Shore	49.4% (9.9%)	54.6% (8.0%)	6.32%	1.81E-02	0.019%	4.27E-02
cg18239431	8	EBF2	Body	North Shore	16.9% (4.7%)	14.7% (3.6%)	–2.49%	2.96E-02	–0.006%	1.75E-01
cg05837727	11			Island	9.3% (2.1%)	8.7% (1.8%)	–1.10%	1.73E-02	–0.004%	1.16E-02
cg18633600	12	LRTM2	Body	Open Sea	28.2% (3.3%)	27.2% (3.1%)	–1.71%	5.97E-02	–0.006%	4.09E-02
cg23350274	14	RDH12	5'UTR	Open Sea	88.6% (1.5%)	88.3% (1.7%)	–1.48%	3.91E-03	–0.005%	9.55E-03
cg21516291	20	SLC35C2	Body	Open Sea	55.7% (4.3%)	53.9% (4.7%)	–2.35%	2.46E-02	–0.007%	5.25E-02

Models adjusted for age, urinary creatinine, BMI, and estimated white blood cell proportions.

### Age-Associated Methylation in Study Population

**Figure 3:**
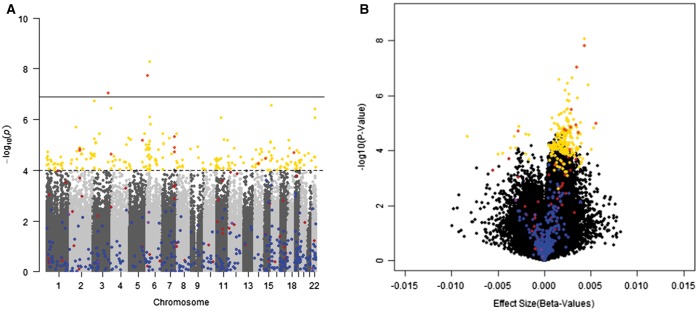
distribution of age-associated markers in study population. **(A)** Manhattan plot of age-associated methylation markers in study population adjusted for BMI, urinary creatinine, and estimated white blood cell composition. Solid line denotes experimental epigenome-wide significance (*P* < 10e^-7^). Dashed line denotes nominal significance level, *P* < 10e-4. **(B)** Volcano Plot of adjusted age-associated markers. For both plots, blue denotes markers included in Horvath predictor, red denotes markers in Hannum predictor, yellow are those significant in our population, orange is the overlap between Hannum and our markers, and purple is the overlap between Horvath and Hannum

We then took an agnostic approach to identify age-associated CpG sites in our study population. In total 114 sites were significantly correlated with age at a significance level of *P* = 0.0001. The absolute values of the Spearman correlations ranged from 0.58 to 0.77. Of the 114 significantly correlated sites, 92% (*n* = 105) were positively correlated with age. After adjusting for BMI, creatinine, and estimate white blood cell proportion, 213 sites were associated with age at a significance level of *P* = 0.0001, and 10 of these sites had an false discovery rate (FDR) less of 0.05 (Fig. 3). Of these sites, 38% (*n* = 43) were among the identified CpG sites correlated with age in our study population (*P* < 1e-4), suggesting that some of these sites were confounded by BMI, urinary creatinine, or white blood cell composition. Ninety-four percent of these sites were positively associated with age. Fifty-four percent of these sites were located within CpG islands and 23% were located within the open sea. This distribution significantly differed from the locational distribution of the entire array (*P* < 0.0001). One hundred sixty sites annotated to a gene. Among these age-associated sites in our population, ten sites were located in the *PRRT1* gene, three sites were located in each of the following genes: *KLF14*, *ELOVL2*, and *FBLN2*.

Among the age-associated sites identified in our study population, 12 sites were nominally significant (*P* < 0.05) with Cd exposure group after adjustment (Table 2). Ten sites were associated with decreased methylation in the HE compared with LE group (range: −1.1 to -2.8%). Two sites were associated with increased methylation between the groups; *PBSM9/TAP1* methylation was 6.4% higher in the HE compared with LE group. Seven sites were associated (*P* < 0.05) with continuous Cd exposure after adjustment.

## Discussion

We observed that the Hannum and Horvath DNAm-ages were correlated with chronologic age in our Cd-exposed study population from northern Thailand and the difference between chronologic age and DNAm-age was smaller in the HE group. Age-associated CpG sites in the Horvath and Hannum were not strongly associated with chronologic age in our study. We identified a unique set of age-associated CpG sites in our study population, suggesting that age-associated methylation patterns vary between populations, by environmental exposures, like Cd, or both. Cd was associated with methylation at age-associated sites included in the Hannum and Horvath DNAm-age predictors, which may affect the estimation of DNAm-age in our study population.

We identified age-associated sites that were unique to our study population, and some of these sites overlapped with those sites included in the Hannum or Horvath DNAm-age predictors, providing support that our results are consistent with age-associated methylation changes identified in the literature [4, 7, 24].Ten age-associated sites in our study population were located within *proline-rich transmembrane protein 1* (*PRRT1*) gene. *PRRT1* lies within the gene dense region of 6p12.32 and is near the major histocompatibility complex, an epigenetically heritable region [25] that encodes numerous immune-response genes, which are susceptible to changes in methylation [26, 27]. Although Hannum and Horvath DNAm-ages correlated with chronologic age, these correlations were stronger in the HE compared with the LE group. Chronologic age correlated more strongly with Hannum DNAm-age compared with Horvath DNAm-age. These DNAm-ages were generated from different tissues, populations, and epigenome-wide arrays, and only six sites overlapped between the Hannum and Horvath DNAm-age predictors. Although the Δage was smaller in the HE group, the mean difference between DNAm-age and chronologic age was positive in both exposure groups, suggesting DNAm-age, a biologic age biomarker, was greater than chronologic age. We postulate that the epigenomic effects of Cd may be different and potentially independent of the epigenomic effects of aging. Within the study, HE women have lower methylation at sites in the Hannum and Horvath predictors compared with LE women. These sites are locationally biased towards promoters and genic regions, which tend to become more methylated during aging, and a majority of these sites have positive coefficients in the predictors [1, 4]. There is a complex relationship among epigenetic modification associated with environmental exposures, age-related processes, and their combined effect on epigenome-wide methylation. Smoking extensively modifies gene-specific and global DNA methylation [28], but it has not been shown to be associated with epigenetic age acceleration [9, 29]. Ambient particle exposure from air pollution is associated with epigenome-wide changes in methylation [30] and is significantly associated with increasing predicted DNAm-age [31].

The epigenomic effects of Cd have not been extensively studied in populations with high environmental exposure, as documented in the Mae Sot district in northern Thailand, and among adults. Two epigenome-wide array studies of Cd have examined cord blood methylation of infants from Bangladesh [32] and USA [33], and both of these studies observed sex-specific associations with maternal blood and placental Cd exposure, respectively. Using a promoter-specific epigenome-wide array, maternal blood Cd altered DNA methylation in promoter regions of 61 genes in infant blood, and some of these genes were associated with transcriptional regulation and apoptosis [34]. Increased urinary Cd exposure in low-exposed Argentinean women was associated with decreased LINE-1 methylation, a surrogate measure of global methylation [35]. Within the Mae Sot population, LINE-1 methylation was lower in HE women compared with HE men, and HE women had decreased MGMT methylation, a methyltransferase that maintains genomic stability, compared with LE women [18]. Cd modifies gene-specific and global methylation, suggesting age-associated methylation changes may be influenced by Cd exposure.

The small sample size of this pilot study limits the interpretation of these results, and our results should be validated longitudinally, in a larger sample size from our study population, or in a different Cd-exposed population, in order to further investigate age-associated methylation changes, epigenetic aging, and Cd exposure. The 450K array only covers a fraction of the total CpG sites in the genome and is biased towards sites located in promoter and intergenic regions. Age-associated methylation changes can only be assessed cross-sectionally in this study, and it is important to examine these changes temporally across the Cd-exposure and age trajectory. Our study only included women because they were mostly non-smokers, and most men in this population smoked. Although environmental tobacco smoke exposure is not likely to contribute substantially to Cd exposure in these women, it could be a source of residual confounding in the analyses. Men and women have different epigenetic aging [4] and Cd exposure patterns [23], and it is likely not appropriate to combine the genders in epigenetic studies of aging. The study population may not be a healthy population since there is a high prevalence of the renal effects associated with Cd exposure in the Mae Sot population. DNAm-ages may accurately estimate the DNAm-age within our study population because the effects of aging and Cd exposure on methylation both change temporally. Other biologic age biomarkers that are more independent of Cd exposure are necessary to validate the association between Cd and biologic aging, and these include transcriptomic age [36], composite biologic age constructed from biomarkers associated with health and function of different organ systems [37], or telomere length [38].

Our study population is an ideal population to examine environmental Cd exposure and age-associated methylation because these women have been environmentally exposed to high levels of Cd and were self-reported non-smokers. Smoking may confound many analyses of environmental exposure to Cd [39]. Urine is also a good biomarker of prolonged Cd exposure as it captures between 10 and 30 years of exposure [40]. Although this pilot study is limited by its sample size and hence power, a concern our study addresses is that there appears to be some population-level heterogeneity in age-associated CpG sites, and the environment may shape aging in a population-specific manner, even though the subjects lived in adjacent villages in northern Thailand. Age-associated epigenetic patterns may be influenced by a combination of different genetic, dietary, lifestyle and environmental factors, and it will be beneficial to develop a biologic age predictor in this population and compare to Horvath and Hannum epigenetic age predictors.

This preliminary study suggests that Cd exposure is associated with age-associated methylation changes although the consequences of these changes are not clear. Biologic age biomarkers may be an important mediator to consider when examining the relationship between environmental Cd exposure and risk of age-related chronic diseases and mortality. The complex interaction among Cd exposure, aging, and methylation may promote a complex chronic disease phenotype that may have long-term consequences during the lifespan. Age-associated methylation changes and their potential modification by Cd suggest that adulthood is another critical window of vulnerability to environmental insults.

## Methods

### Study Population

Forty non-smoking women between ages 40 and 80 years were selected from a Cd-exposed cohort from Mae Sot, Thailand. These women participated in a follow-up health impact survey in 2012 and provided whole blood and urine at the time of the survey [41].This Cd-exposed cohort has been previously described in [20], and all covariates included in the analyses were obtained from the health impact survey and biomarkers of renal functions were previously measured [42]. We selected non-smoking women from the population analyzed by Virani *et al*. [18]. Based on their urinary Cd adjusted by specific gravity, we identified 20 women with HE from the highest quartile, and twenty women with LE from the lowest quartile. Women in the LE group had Cd exposure <2 µg/l, which corresponds to urinary Cd concentrations in humans not excessively exposed to Cd [43]. The women in each group were matched by age within 5 years. Study approval for this study was obtained from the research and ethics committee from the Faculty of Medicine, Chiang Mai University (Approval No. 004/2012).

### Cd Exposure Assessment

Twenty-five milliliters of morning urine were collected in Cd-free polyethylene containers. Specific gravity (SG) was measured for each sample using a refractometer (PALS-10S), shortly after collection. The median specific gravity in the study population in 2012 was 1.015, and we used the following adjustment, unadjusted Cd (µg/l) * [(1.015–1)/(SG-1)], to account for urine density at time of collection [44]. After transportation to the University of Michigan on dry ice, samples were stored at −20 °C until analysis. Urinary Cd was measured at the Michigan Department of Community Health. The samples were diluted 1:10 with a digestion solution of 2.0% nitric acid, 0.05% Triton X, and internal standards. Cd concentrations were measured using an inductively coupled plasma mass spectrometer. The analytical accuracy using Cd urinary standard reference material (QMEQAS08U, Institut National de Santé Publique du Québec) was 101.1% (*n* = 8), and all samples were above the detection limit of 0.15 µg/l.

### DNA Extraction and Bisulfite Conversion

Five milliliters of fasting venous whole blood was collected in EDTA-coated tubes. After transportation to the University of Michigan on dry ice, samples were stored at −80 °C until DNA extraction. Genomic DNA was extracted from 300 µL of whole blood using QiaAMP DNA Mini Kit (Qiagen). Extracted DNA concentrations and quality were quantified using the Nanodrop spectrophotometer (Thermoscientific). We bisulfite converted 500 ng of genomic DNA using EpiTect Bisulfite Kit (Qiagen) per manufacturer’s protocol.

### DNA Methylation Measurement

DNA methylation was measured using the Illumina Infinium HumanMethylation450 BeadChip [45]. Human Illumina 450K Methylation arrays were processed by the University of Michigan Sequencing Core Facility according to manufacturer protocol. The proportion of methylation at each site is reported as the beta-value, which is computed as beta = methylated signal intensity/(methylated signal intensity + unmethylated signal intensity + a), where a is an adjustment constant.

### Normalization Summary

We filtered 1977 probes with detection *P*-values above 0.01 and 3198 probes with bead counts < 3 in two or more samples from the analysis. We restricted our analysis to probes corresponding to CpG sites located on autosomal chromosomes and removed 11 270 probes on the sex chromosomes. No samples in our analysis performed poorly, as defined by having >5% of probes above the detection *P*-value. We performed background and dye-bias correction using the normal-exponential model [46] implemented in methylumi [47]. We also removed probes identified by Chen *et al*. [48], where the CpG site or single-base extension site corresponded to a SNP with a minor allele frequency ≥ 0.01, which removed 24 720 probes, and 29 233 cross-reactive probes. We then applied subset-quantile within array normalization [49]. ComBat was used to correct for plate and technical effects [50, 51]. The final number of probes included in our analysis was 419 995, which covers 80% of the 450K Illumina array.

### Age Prediction

For the age prediction analyses, we generated background corrected betas. To estimate predicted methylation age (DNAm-age), we applied two published methods from Hannum *et al*. [4] and Horvath [1]. Hannum *et al*. [4] found that a combination of 71 age-associated methylation markers predicted the age of individuals ages 9–101, and additionally, identified individuals whose DNAm-age significantly deviated from their chronologic age. Horvath developed a predictive model for aging utilizing data from 51 tissues and cell types and identified a panel of 353 age-associated methylation markers that he proposed estimated the epigenetic clock and DNAm-age within an individual [1]. Horvath DNAm-age was computed using his online age calculator (https://dnamage.genetics.ucla.edu). Hannum DNAm-age was computed by extracting the effect for each age-related CpG site in the predictor and weighting our betas with each site. The sum of these weights yielded the Hannum DNAm-age. DNAm-ages were adjusted by array and estimated Houseman white blood cell composition using linear regression [1, 52], and the residuals from this model were added to the mean DNAm-age to yield an adjusted DNAm-age [9]. We calculated epigenetic age acceleration as the difference between adjusted DNAm-age and chronologic age (Δage).

### Statistical Analysis

All statistical analyses were performed on the *M*-values from the beta-values using a logit2 transformation. We examined the unadjusted age associations by computing the Spearman correlation for each CpG site and adjusted using linear multivariate regression performed in *limma*. Based on review of epigenome-wide analysis literature, we selected covariates *a priori* and adjusted for BMI, urinary creatinine, as a marker of renal function, and estimated white blood cell proportions using the Houseman method [52]. Differences in methylation between Cd exposure groups and log-transformed Cd were analyzed using linear mixed models among the age-related sites in our study population, reported by Hannum *et al.* [4] and Horvath [1] using *lmerTest.* The relationship between DNAm-age and chronologic age were examined using Spearman correlations and linear models, and differences in Δage were examined using linear mixed models, described earlier. The FDR was computed using the Benjamini-Hochberg method. All analyses were implemented in R version 3.2.3.

## Funding

Support for this study was provided by the University of Michigan School of Public Health Office of Global Public Health and the Center for Southeast Asian Studies.
